# New data on karyotype, spermatogenesis and ovarian trophocyte ploidy in three aquatic bug species of the families Naucoridae, Notonectidae, and Belostomatidae (Nepomorpha, Heteroptera)

**DOI:** 10.3897/CompCytogen.v14i1.48709

**Published:** 2020-03-05

**Authors:** Desislava Stoianova, Nikolay Simov, Manh Quang Vu, Duc Minh Nguyen, Snejana Grozeva

**Affiliations:** 1 Institute of Biodiversity and Ecosystem Research, Bulgarian Academy of Sciences, 1 Tsar Osvoboditel Blvd., Sofia 1000, Bulgaria; 2 National Museum of Natural History, Bulgarian Academy of Sciences, 1 Tsar Osvoboditel Blvd., Sofia 1000, Bulgaria; 3 Hanoi National University of Education (HNUE), 136 Xuan Thuy Rd., DHSP Cau Giay; c/o Ho Chi Minh City University of Food Industry, 140 Le Trong Tan St., Tan Phu, Ho Chi Minh City, Vietnam; 4 Institute of Ecology and Works Protection, Hanoi, Vietnam

**Keywords:** achiasmate male meiosis (achiasmy), B-chromosomes, karyotype, nurse cells, South Europe and Southeast Asia, *Diplonychus rusticus*, *Ilyocoris cimicoides*, *Notonecta glauca*

## Abstract

We report the karyotype, some aspects of spermatogenesis, and ovarian trophocytes ploidy in three aquatic bug species: *Ilyocoris cimicoides* (Linnaeus, 1758), *Notonecta glauca* Linnaeus, 1758, and *Diplonychus rusticus* Fabricius, 1871 from previously unexplored regions – South Europe (Bulgaria) and Southeast Asia (Vietnam). Our results add considerable support for the published karyotype data for these species. In *I. cimicoides*, we observed achiasmate male meiosis – the first report of achiasmy for the family Naucoridae. More comprehensive cytogenetic studies in other species of the Naucoridae are required to elucidate the role of achiasmy as a character in the systematics of the family.

Our observations on the association between phases of spermatogenesis and developmental stages in *I. cimicoides* and *N. glauca* differ from the previously published data. In these species, we assume that the spermatogenesis phases are not strongly associated with certain developmental stages. For further cytogenetic studies (on the Balkan Peninsula), we recommend July as the most appropriate month for collection of *I. cimicoides* and *N. glauca*.

In the ovaries of both species, we studied the level of ploidy in metaphase and interphase trophocytes. In *I. cimicoides*, diploid and tetraploid metaphase trophocytes were found. Heteropycnotic elements, observed in interphase trophocytes of this species, represented the X chromosomes. It allowed us to determine the trophocytes ploidy at interphase (2n was repeated up to 16 times). The situation with *N. glauca* was different. The metaphase trophocytes were diploid and we were not able to determine the ploidy of interphase trophocytes since such conspicuous heteropycnotic elements were not found. The scarce data available suggest a tendency for a low level of trophocyte ploidy in the basal infraorders (Nepomorpha and Gerromorpha) and for a high level in the more advanced Pentatomomorpha. Data about this character in species from other infraorders are needed to confirm that tendency.

## Introduction

*Ilyocoris cimicoides* (Linnaeus, 1758), *Notonecta glauca* Linnaeus, 1758, and *Diplonychus rusticus* Fabricius, 1871 are common predators in freshwater basins. The first two species are broadly distributed across the Palearctic region. *Ilyocoris cimicoides* (Naucoridae) inhabits most of Europe and Asia from Anatolia to Siberia and North China (Fent et al. 2011). *Notonecta glauca* (Notonectidae) occurs in Europe, North Africa, western parts of Central Asia, reaching northwest China (Polhemus 1995, Linnavuori and Hosseini 2000, Kanyukova 2006, Fent et al. 2011). *Diplonychus rusticus* (Belostomatidae) is widespread in the warmer regions of India, Sri Lanka, Southeast Asia, Malaysia, Sumatra, Java, Borneo, Sulawesi, Philippines, New Guinea, China, and Japan (Chen et al. 2005, Polhemus and Polhemus 2013). Although these three species have broad ranges, karyotype data for them have been published only from few regions: *I. cimicoides* – northern of the Danube River (Steopoe 1929); *N. glauca* – England (Browne 1916, Angus et al. 2004), Finland (Halkka 1956), Netherlands (Pantel and Sinety 1906), *D. rusticus* – India (Bawa 1953, Jande 1959). A recently described variation in chromosome number between different populations of two broadly distributed species of Nepomorpha (Angus et al. 2017) have raised the question if there are such cytogenetic differences between populations of other broadly distributed species (*I. cimicoides*, *N. glauca*, and *D. rusticus*).

In many insect species, spermatogenesis completes at the final preimaginal developmental stage, so that the testes of adults contain only spermatids/spermatozoa (Dumser 1980, Gillot 1995). In Heteroptera, adults are traditionally used for cytogenetic studies as at this stage, accurate species identification is easy and spermatogenesis still occurs (e.g. Nokkala and Nokkala 1999, Grozeva and Nokkala 2003, Lanzone and Souza 2006, Castanhole et al. 2008, Golub et al. 2018, Grozeva et al. 2019, for extensive bibliography see Papeschi and Bressa 2006). However, in last (V) instar and adults of nepomorphan species *I. cimicoides* and *N. glauca*, testes were shown to contain only spermatids/spermatozoa. Spermatogenesis has been observed only in earlier developmental stages: instar III and IV nymphs of *I. cimicoides* (in Papáček and Gelbič 1989) and instar IV and V of *N. glauca* (Papáček and Soldán 1992). Spermatogenesis was not the focus of the cited studies; the authors’ comments are based only on histological analysis of testes.

A detailed cytogenetic analysis could elucidate the association of certain stages of meiosis with definite instars. Such data would be useful in further cytogenetic studies of these species (e.g. to collect the most appropriate developmental stage with meiotic or mitotic divisions).

In hemipteran species (incl. Heteroptera), the ovaries consist of meroistic telotrophic ovarioles, characterised by a tropharium in the apex and a vitellarium in the basal part (Ma and Ramaswamy 1987). The organisation of the tropharium has been studied in species of each of the infraorders of Heteroptera but Enicocephalomorpha (Eschenberg and Dunlap 1966, Heming-van Battum and Heming 1986, Bilinski et al. 1990, Jawale and Ranade 1990, Simiczyjew et al. 1996, Štys et al. 1998, more references in Štys et al. 1998 and in Simiczyjew et al. 1998). Significant differences in the species of the basal infraorders compared to those of the more advanced infraorders have been found (Simiczyjew et al. 1998). Unlike the organisation of the tropharium, very little attention has been given to the ploidy of trophocytes (nurse cells). The level of trophocyte ploidy in heteropteran ovarioles has been reported only for three species of two infraorders. Choi and Nagl (1977) measured diploid DNA content increase in trophocytes of *Gerris najas* (De Geer, 1773) – 16-fold increase; Cave (1975) – in *Oncopeltus fasciatus* (Dallas, 1852) – 128-fold increase; Dittmann et al. (1984) in *Dysdercus intermedins* Distant, 1902) – 124-fold increase. The first species (*G. najas*) belongs to one of the basal (Gerromorpha), while the last two species – to one of the most advanced (Pentatomomorpha) infraorders. In the ovaries of insects of another hemipteran group – aphids, the level of trophocyte ploidy is suggested to be species specific (Michalik et al. 2013), but for the true bugs there is no confirmation of that.

The aim of the present cytogenetic study was to examine *Ilyocoris cimicoides*, *Notonecta glauca*, and *Diplonychus rusticus* originating from previously unexplored regions – South Europe (Bulgaria) and Southeast Asia (Vietnam), in order to 1) check cytogenetic differences between populations; 2) analyse the relationship between the developmental stages and the phases of spermatogenesis in testes for *I. cimicoides* and *N. glauca*; and 3) determine the ploidy level of trophocytes in ovaries on the example of *I. cimicoides* and *N. glauca*.

## Material and methods

**Figure 1.**
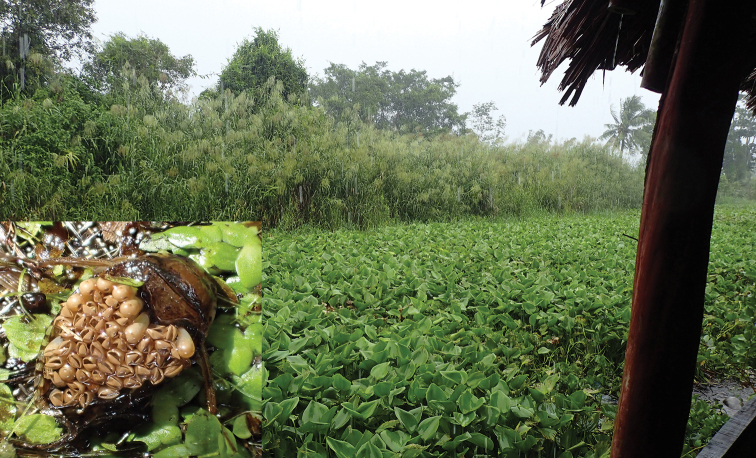
*Diplonychus rusticus*, male carrying eggs and typical habitat of the species in U Minh Ha National Park, Ca Mau Province, Vietnam.

Specimens of *Diplonychus rusticus* (Belostomatidae) (4 males) were collected in September 2018 from Vietnam: Ca Mau Province, Tran Van Thoi District, Tran Hoi commune, U Minh Ha National Park, 09.22521N, 104.95898E (Fig. 1). Specimens of *Ilyocoris cimicoides* (Naucoridae) (16 males, 7 females) and *Notonecta glauca* (Notonectidae) (19 males and 6 females) were collected during the period September 2018-August 2019 from three different localities in Bulgaria: Pernik Province, Choklyovo blato Marsh, 42.40252N, 22.82234E; Sofia Province, artificial pond in a park in the City of Sofia, 42.66355N, 23.30742E; Sofia Province, pools near the Town of Ihtiman, 42.459837N, 23.804966E. For cytogenetic studies, the insects were fixed in the field in 3:1 fixative (96% ethanol: glacial acetic acid). The gonads were dissected out and squashed in a drop of 45% acetic acid. The coverslips were removed using dry ice. Slides were dehydrated in fresh fixative (3:1) and air-dried. The preparations were stained using the Schiff-Giemsa (Grozeva and Nokkala 1996) and C-banding (Grozeva et al. 2004) method. The chromosomal location of 18S rDNA clusters was determined by the well-known FISH protocol (Grozeva et al. 2011, 2015, 2019, Golub et al. 2019).

Giemsa stained preparations were analysed under an Axio Scope A1 – Carl Zeiss Microscope) at 100× magnification and documented with a ProgResMFcool – Jenoptik AG digital camera. FISH preparations were analysed under a Leica DM 6000 B microscope and images were acquired using a Leica DFC 345 FX camera and Leica Application Suite 3.7 software with an Image Overlay module.

The specimens and the chromosome preparations used for this study are stored at the Lab of Cytotaxonomy and Evolution, Institute of Biodiversity and Ecosystem Research, BAS (Sofia, Bulgaria).

## Results and discussion

### Description of the karyotype

***Ilyocoris cimicoides*, 2n = 51 (48A + 2m + X) ♂**

The morphology of the testes of the examined males and the ovaries of the females matched the descriptions given by Papáček and Gelbič (1989) and Papáček et al. (1997), respectively.

**Figures 2–10.**
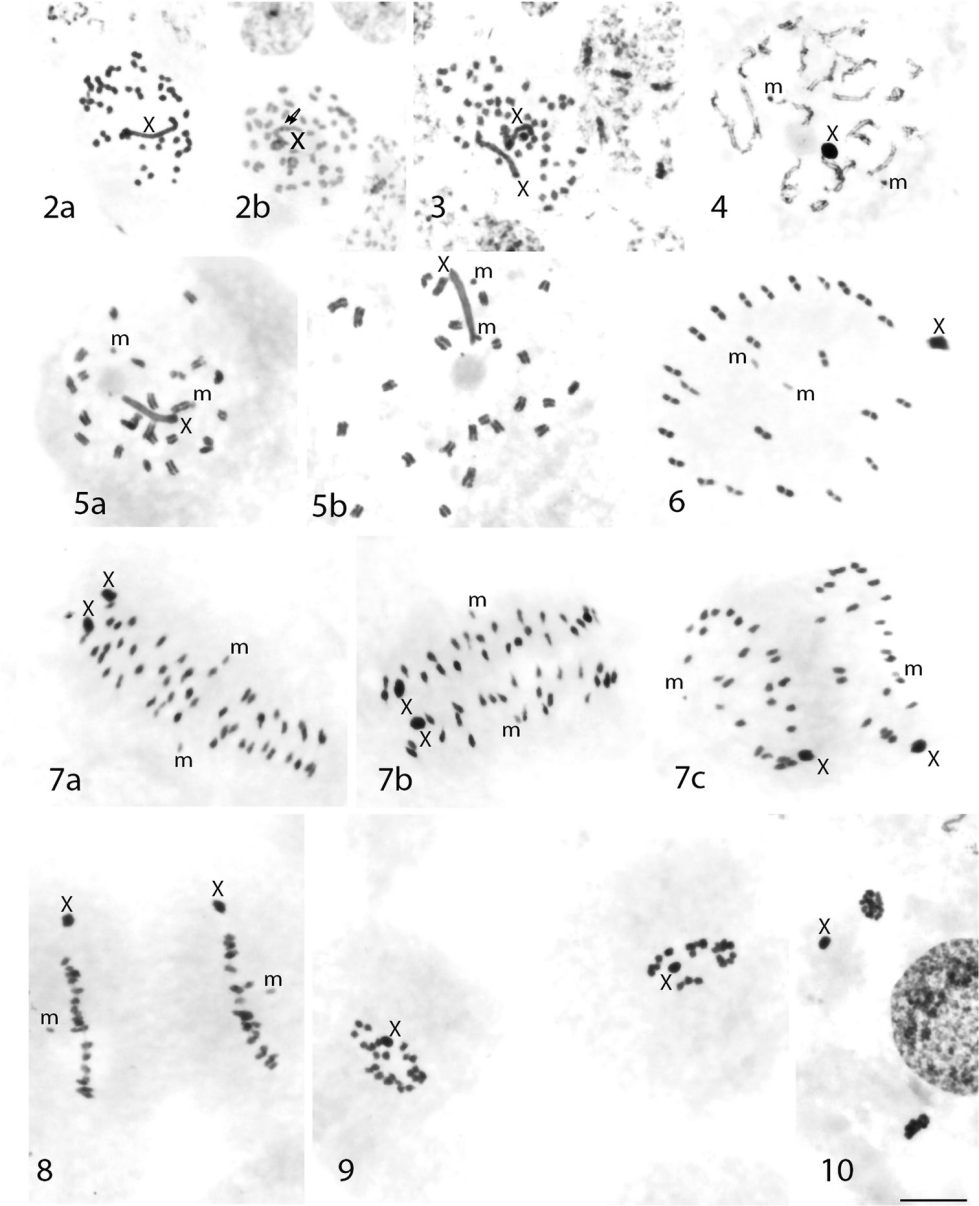
*Ilyocoris cimicoides* (testis/ovary): Schiff-Giemsa (**2a, 3–10**), C-banding (**2b**) **2a, b** spermatogonial metaphase (arrows indicate heterochromatin blocks) **3** oogonial metaphase **4–8** primary spermatocytes: **4** early condensation stage **5a, b** late condensation stage **6** metaphase I **7 a–c** anaphase I **8** telophase I **9, 10** secondary spermatocytes: **9** metaphase II **10** two telophases II – one with X, another without X. Scale bar:10 μm.

Like all heteropteran species (Ueshima 1979, Papeschi and Bressa 2006, Kuznetsova et al. 2011), the chromosomes of this species are holokinetic – without localized centromere. In studied male and female nymphs, mitotic metaphases consisted of 48 autosomes (Figs 2 a, b, 3). Except the 48 autosomes, the karyotype included a pair of very small m-chromosomes (see below the description of the meiotic metaphase I), difficult for observation in mitotic cells. The X chromosomes were the largest chromosomes of the complement. At spermatogonial metaphase, the X chromosome displayed interstitial heterochromatin blocks after C-banding (Fig. 2b). In the early condensation stage (late meiotic prophase), we observed 24 bivalents consisting of two side-by-side aligned chromosomes without any sign of chiasmata between them, a pair of m-chromosome univalents, and the heteropycnotic X chromosome, which usually appeared close to the nucleolus (Fig. 4). At the late condensation stage, the X still tended to be close to the nucleolus (Fig. 5a, b). At metaphase I (MI), the autosomal bivalents were similar in size. Most of them formed a ring. The m-chromosome pair and some of the autosomal bivalents laid inside the ring (Fig. 6). The X chromosome was usually seen close to the periphery of the ring. The post-reduction for the sex chromosomes in male meiosis is another specific cytogenetic character of Heteroptera being typical for the majority of the studied species and higher taxa of this group (Ueshima 1979, Kuznetsova et al. 2011). Such was the case in *I. cimicoides*: the X was observed in all the examined anaphase I (AI) (Fig. 7 a–c), telophase I (TI) nuclei (Fig. 8) and in all daughter cells at metaphase II (MII) (Fig. 9). At AI, it was easy to distinguish the m-chromosomes, which were going ahead of the set (Figs 7, 8). At MII, only the large X could be recognized reliably (Fig. 9). At telophase II (TII), the second (equational for the autosomes but reductional for the sex chromosomes) division resulted in two types of daughter cells – with and without X chromosome (Fig. 10).

**Figures 11–18.**
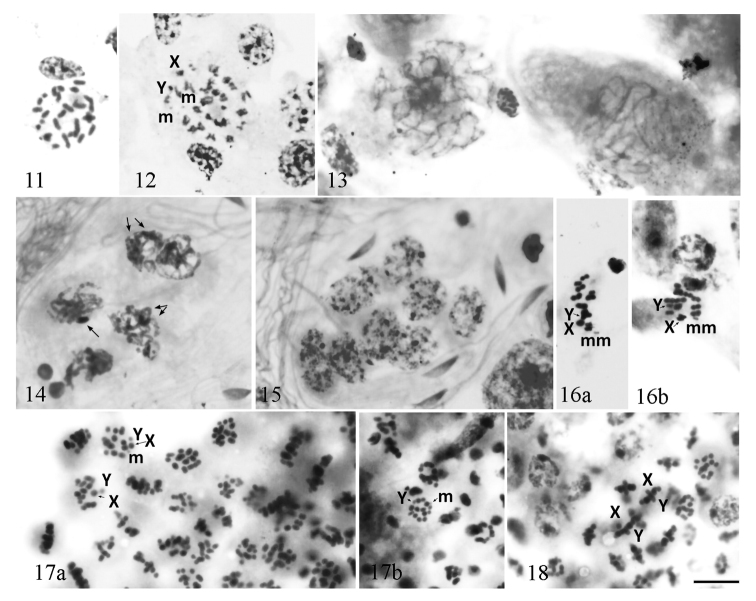
*Diplonychus rusticus* (testis) **11** spermatogonial metaphase **12** spermatogonial anaphase **13–16** primary spermatocytes: **13** leptotene **14** pachytene **15** diffuse stage **16a, b** metaphase I **17, 18** secondary spermatocytes: **17 a, b** metaphase II **18** anaphase II. Scale bar:10 μm.

Cytogenetic data on *I. cimicoides* have been published by Divaz (1915) and Steopoe (1929). Divaz (1915) studied the spermatogenesis of *I. cimicoides* from Serbia, but he did not actually deal with the karyotype: he focused on the presence and behaviour of specific chromatophilic bodies (“corpuscules archoplasmiques”). Our results confirmed the chromosome formula of 2n = 51 (48A + 2m + X), and the chromosomes behaviour for male *I. cimicoides* reported by Steopoe (1929), based on specimens (without information of their developmental stage) collected north of the Danube River. These authors did not mention anything about the formation of chiasmata. In the present study, we provide the first report for achiasmate male meiosis in *I. cimicoides* and in the whole family Naucoridae. The achiasmate type of meiosis, called also “achiasmy” (Satomura et al. 2019), as a rule, is restricted to the heterogametic sex (White 1973). Hitherto, achiasmate male meiosis has been found in eight other families of three Heteroptera infraorders, namely Cimicomorpha: Anthocoridae, Microphysidae, Cimicidae, Miridae, and Nabidae; Leptopodomorpha: Saldidae; Nepomorpha: Micronectidae and Corixidae (Stoianova et al. 2015, more references in Kuznetsova et al. 2011). Most of the studied families seem to be homogenous in respect to the presence/absence of chiasmata. Based on the cited data it is suggested that in Heteroptera achiasmy is a stable cytogenetic characteristic at the family level (Grozeva et al. 2008, Kuznetsova et al. 2011). However, heterogeneity in respect to this character has been observed in family Corixidae (Nepomorpha) (Stoianova et al. 2015). Achiasmy is reported for two *Cymatia* species, while the rest examined species of the Corixidae display chiasmata. The achiasmy in *I. cimicoides* (present study) reveals the heterogeneity of the Naucoridae in respect to this character. Hitherto, cytogenetic studies have been published for eight other naucorid species (Papeschi 1992, for more references see Ueshima 1979). Two of them, *Pelocoris lautus* Berg, 1879 and *P. binotulatus* (Stål, 1862), were shown to display chiasmate meiosis in males (Papeschi 1992), while for another six species no information on the presence/absence of chiasmata has been provided (for references see Ueshima 1979). It is noteworthy that in another insect order (Diptera) heterogeneity in the type of male meiosis (achiasmate/chiasmate) has been reported even at genus level (see Satomura et al. 2019). Taking into consideration these new findings, more comprehensive cytogenetic studies in other species of the family Naucoridae are required to elucidate the type of meiosis (chiasmate/achiasmate) as a character in the systematics of the family.

### *Diplonychus rusticus*, 2n = 28 (24A + 2m + XY) ♂

The internal reproductive system of the examined adult males confirmed the morphological descriptions given for *Diplonychus rusticus* by Pendergrast (1957: as *Sphaerodema rusticum* Fabricius, 1871). Every colorless testis consisted of one more or less spherical follicle, decreasing in diameter from the apex to the vas deferens, which expanded to a vesicula seminalis. Such structure of the male reproductive system was described and illustrated well in another Belostomatidae species – *Lethocerus patruelis* (Stål, 1854) (Grozeva et al. 2013).

Chromosome complement in males of *D. rusticus* (as *S. rusticum*) from India was published as 2n = 28 (24A + 2m + XY), together with drawings of the chromosomes at different stages of spermatogenesis (Bawa 1953, Jande 1959). These authors claim a symmetric karyotype and describe in detail all stages of spermatogenesis: spermatogonial mitosis and the behaviour of the chromosomes during both meiotic divisions.

We studied males of this species from Vietnam, collected in U Minh Ha National Park. Spermatogonial metaphases resembled those of *D. rusticus*, *D. annulatus* (Fabricius, 1781) and *D. molestus* (Dufour, 1863) (as *D. subrhombeus* (Mayr, 1871)) studied from India (Bawa 1953, Jande 1959). They consisted of 28 chromosomes (Fig. 11) but it was difficult to identify individual chromosomes in the set. At early spermatogonial anaphase, the chromosomes were split in chromatids, lying in parallel (Fig. 12), which is the case for holokinetic chromosomes (for instance, Lukhtanov et al. 2019) and one could recognize the X, Y and m-chromosomes. At leptotene (Fig. 13), long and thin chromosomes began to thicken, the sister chromatids were not visible as separate entities. At pachytene (Fig. 14), one or two heteropycnotic bodies of the sex chromosome heterochromatin could be observed. During the diffuse stage, the sex chromosomes were more often associated with each other (Fig. 15). At MI, 12 bivalents, two sex chromosomes as univalents, and a pseudobivalent of the m-chromosomes could be seen (Fig. 16 a, b). At MII, every plate consisted of 12 autosomes, a pseudobivalent of the sex chromosomes, and one m-chromosome (Fig. 17 a, b). The first meiotic division was thus reductional for the autosomes and equational for the sex chromosomes. At AII, the sex chromosomes were going ahead to the poles (Fig. 18). The study of males from Vietnam fully confirmed the observations and description on the spermatogenesis of this species from India (Bawa 1953, Jande 1959). Here, we provide for the first time, photographs of the spermatogenesis stages for *D. rusticus*.

### *Notonecta glauca*, 2n = 24 (20A + 2m + XY) ♂

The ovaria of the females and the testes of the males examined matched the morphological descriptions given by Papáček and Soldán (1987, 1992, respectively).

**Figures 19.**
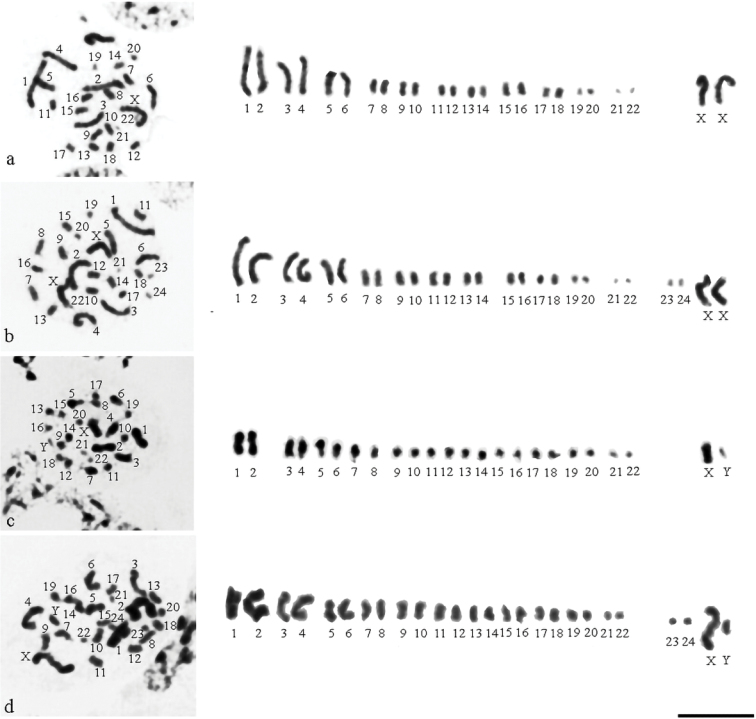
*Notonecta glauca* (testis/ovary) **a, b** mitotic metaphase in a ovariole: **a** with 24 chromosome elements **b** with 26 chromosome elements **c, d** mitotic metaphase in a testis: **c** with 24 chromosome elements **d** with 26 chromosome elements. Scale bar:10 μm.

In gonads of females and males, we found mitotic metaphase plates with 24 chromosomes including two sex chromosomes (Fig. 19 a, c). As an exception, only two mitotic metaphase plates with 26 elements were found: one in a ovariole (Fig. 19 b), in which we found mitotic metaphase plates with 24 chromosomes and one in a testis (Fig. 19 d), in which we found mitotic metaphase plates with 24 chromosomes.

**Figures 20–23.**
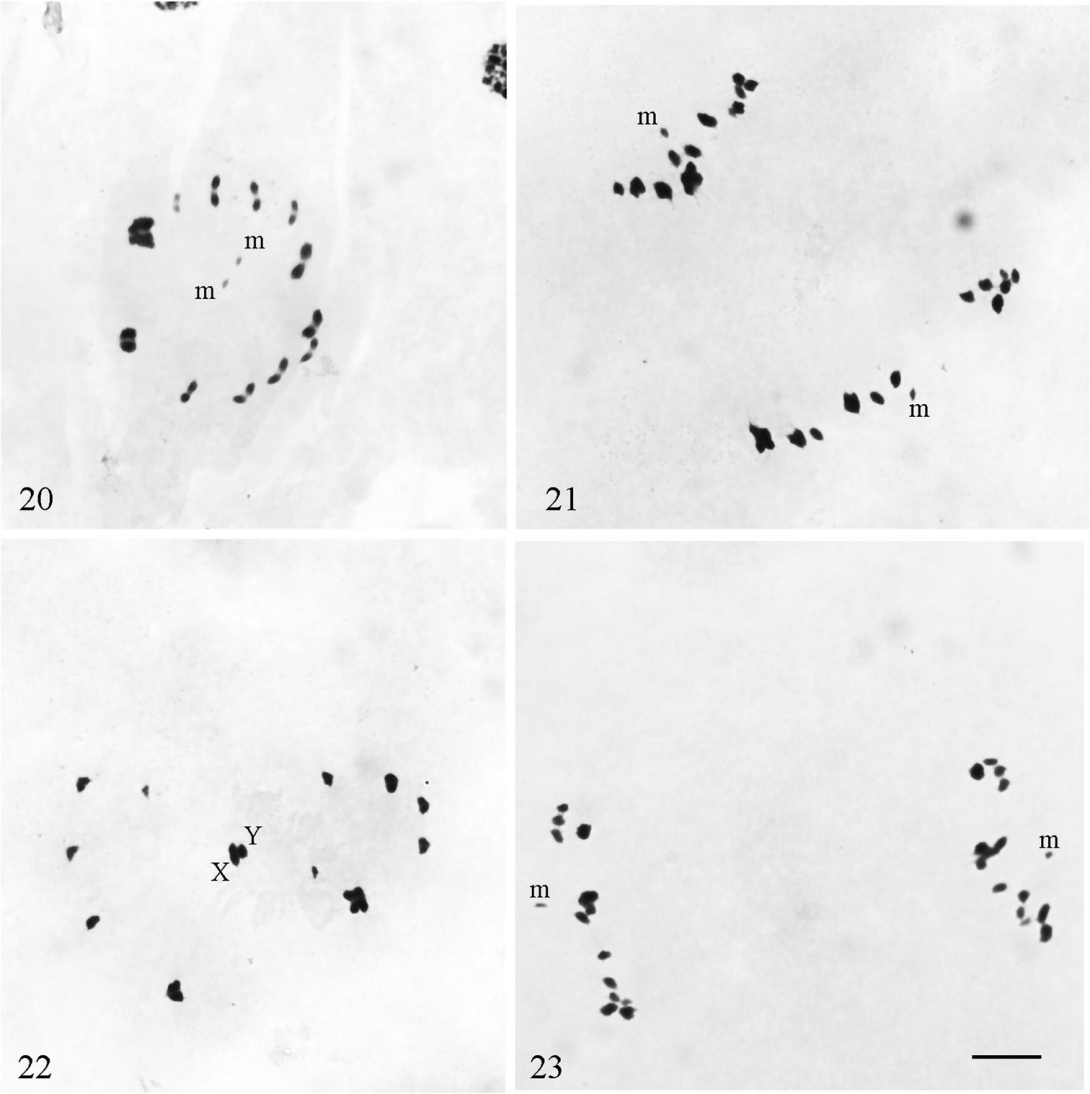
*Notonecta glauca* (testis/ovary) **20, 21** primary spermatocytes: **20** metaphase I **21** anaphase I **22, 23** secondary spermatocytes: **22** metaphase II with 13 chromosome elements **23** anaphase II. Scale bar:10 μm.

In adults collected in July, we found the advanced stages of spermatogenesis, from MI (Fig. 20) to AII (Fig. 23). At MI, 10 autosomal bivalents and the X and Y chromosomes formed a ring with a pseudobivalent of m-chromosomes in it (Fig. 20). It was difficult to distinguish the sex chromosomes in the majority of the meiotic stages. At all the examined AI nuclei, 13 chromosome elements could be counted with the m-chromosomes going ahead of the set (Fig. 21). At MII, a pseudobivalent of the sex chromosomes was placed inside a ring formed by the autosomes; the m-chromosome was indistinguishable (Fig. 22).

Our observations confirm the chromosome formula of 2n = 24 (20A + 2m + XY) and post-reduction of the sex chromosomes reported by Browne (1916) and Halkka (1956) for males of *N. glauca*, collected in England and Finland, respectively. Angus et al. (2004) studied the karyotype of *N. glauca* in midgut cells from both male and female specimens collected in England. He found some specimens with 2n = 26 along with the regular 2n = 24. Our observations of mitotic metaphase plates with 26 chromosomes confirm the occasional occurrence of an extra pair of chromosomes, which was interpreted by Angus et al. (2004) as that of the B-chromosomes. In the present study, C-banding technique was performed but it did not provide additional information.

### Association between phases of spermatogenesis and developmental stages in *Ilyocoris cimicoides* and *Notonecta glauca*

Our observations of the association between phases of spermatogenesis and developmental stages in *I. cimicoides* and *N. glauca* differ from those published for these species by Papáček and Gelbič (1989) and Papáček and Soldán (1992), respectively (see Table 1). In *I. cimicoides*, we observed meiotic divisions in instar V, while Papáček and Gelbič (1989) reported the instar V to have spermatids and spermatozoa only. In adults of *N. glauca*, we observed meiotic divisions, while Papáček and Soldán (1992) found only spermatids and spermatozoa. According to our observations, in both *I. cimicoides* and *N. glauca*, males of the same stage of development show different stages of spermatogenesis if they are collected in different seasons (Table 1). For these species, we assume thus that the spermatogenesis phases are not strongly associated with certain developmental stages. It could be speculated that spermatogenesis phases in these species follow the seasonal changes of factors such as temperature and/or photoperiod. There are no publications about the influence of temperature and/or photoperiod on spermatogenesis in heteropteran species. Nevertheless, the influence of these factors on the development and reproduction of Heteroptera has been well documented (Dunbar and Bacon 1972, Ali and Ewiess 1977, Spence et al. 1980, Braman and Pendley 1993, Nagai and Yano 1999, Niva and Takeda 2003, Zerbino et al. 2013, Gusev and Lopatina 2018, Santos et al. 2018). Temperature effect on the development of subspecies *Ilyocoris cimicoides exclamationis* (Scott, 1874) from Japan has also been reported (Kaneda and Yoshiyasu 2007). We have no data on the environmental factors in the collection sites; therefore, the present study cannot contribute to understanding the influence of environmental factors on the spermatogenesis in the studied species. Nevertheless, for practical purposes, we assume that in Bulgaria (in regions at less than 800 m a.s.l.) and probably on the Balkan Peninsula, July is the most appropriate month for collecting *I. cimicoides* and *N. glauca* for cytogenetic studies (Table 1).

**Table 1.** Stages of spermatogenesis and spermiogenesis observed in the testes of IV and Vth instars and adult of *I. cimicoides* and *N. glauca*.

Developmental stage	*Ilyocoris cimicoides*		*Notonecta glauca*	
	After Papáček and Gelbič (1989)	Present data	After Papáček and Soldán (1992)	Present data
**Instar III**	primary spermatocytes and as result of MI – secondary spermatocytes	spermatogonial stages	primary spermatocytes	spermatogonial stages
**Instar IV**	primary spermatocytes and as result of MI – secondary spermatocytes	spermatogonial stages and meiotic prophase stages	primary spermatocytes and as result of MI – secondary spermatocytes	spermatogonial stages
**Instar V**	collected in the end of the summer – bundles of spermatozoa	collected in July and August – from PMI to telophase II	disappearance “of the zone of spermatogonia” (the end of the spermatogonial divisions)	spermatogonial stages and meiotic prophase stages
		collected in September – spermatids/spermatozoa		
**Adult**	mature spermatozoa	spermatids/spermatozoa	No information on the month of collection – spermatids/spermatozoa	collected in July – from MI to AII
				collected in September – spermatids/spermatozoa

### Trophocyte ploidy in the ovarioles of *N. glauca* and *I. cimicoides*

**Figures 24–28.**
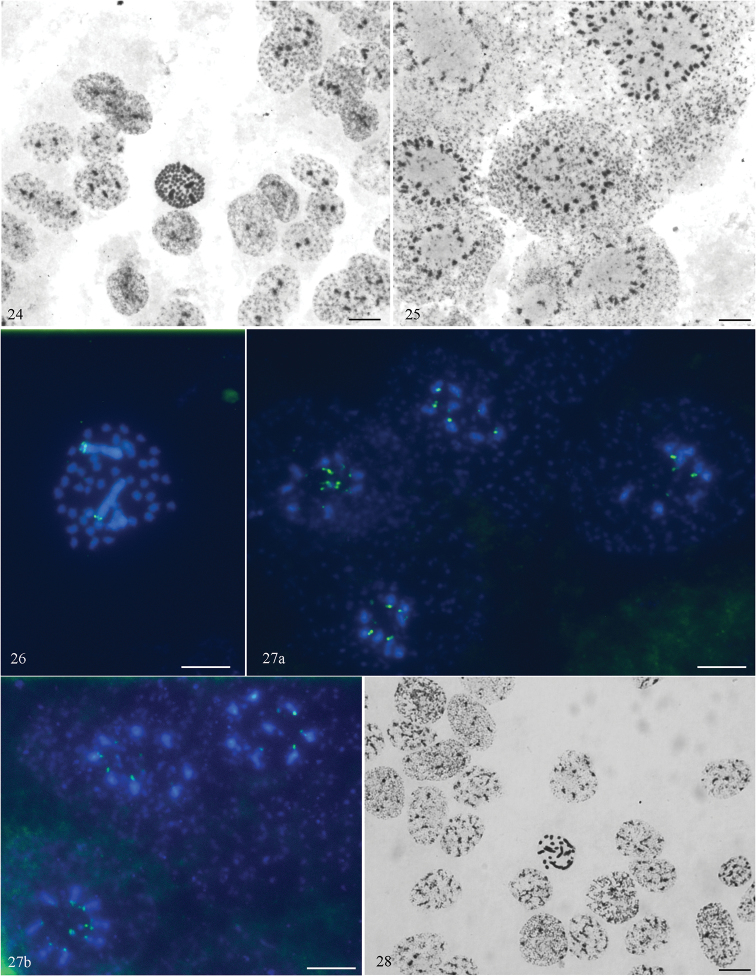
*Ilyocoris cimicoides* and *Notonecta glauca* (ovarioles): Schiff-Giemsa (**24, 25, 28**) and FISH with 18S rDNA (**26, 27**) **24–27***Ilyocoris cimicoides*: **24** interphase trophocytes with 2 and 4 heteropycnotic elements, and a metaphase tetraploid trophocyte **25** interphase trophocytes with 16 and about 32 heteropycnotic elements **26** 18S rDNA signals on the sex chromosomes in a oogonial diploid metaphase plate **27 a, b** 18S rDNA signals on the heteropycnotic elements in interphase octoploid trophocytes **28***Notonecta glauca*: interphase trophocytes and a mitotic metaphase diploid trophocyte. Scale bars: 10 μm.

In the ovaries of *I. cimicoides* and *N. glauca*, we studied the level of ploidy both in mitotic (metaphases) and in interphase trophocytes (nurse cells). Among the trophocytes of *I. cimicoides*, only diploid and tetraploid metaphases were found (Fig. 24). In *I. cimicoides*, we observed interphase trophocytes with conspicuous heteropycnotic elements, which varied in number from 2 to 32 (Figs 24, 25), almost always an even number. In cells with a higher (8–32) number of elements, the exact counting was often impeded by the clumping of the elements. In ovarian mitosis of *I. cimicoides*, hybridization signals after FISH for 18S rDNA were found in the telomeric region of the sex chromosomes (Fig. 26). The same hybridization signals were likewise found in heteropycnotic elements of trophocytes (Fig. 27 a, b). This suggests that the heteropycnotic elements found in the trophocytes represent the sex chromosomes. Thus, in *I. cimicoides* the interphase trophocytes with high number of heteropycnotic elements, i.e. X chromosomes, were highly polyploid (2n was repeated up to 16 times). The situation with *N. glauca* was different. We did not find interphase trophocytes with such conspicuous heteropycnotic bodies (Fig. 28) and we were able to determine the ploidy (2n) only in mitotic trophocytes (Figs 19, 28).

The level of trophocyte ploidy in the ovarioles of *I. cimicoides* (Nepomorpha) (present study) is the same (16 times increase) as reported for *Gerris najas* (Gerromorpha) (Choi and Nagl 1977). Much higher level of trophocyte ploidy has been reported for the two hitherto studied species of infraorder Pentatomomorpha: *Oncopeltus fasciatus* – 128 times increase (Cave 1975); *Dysdercus intermedins* – 124 times increase (Dittmann et al. 1984). The scarce data presently available suggest a tendency for a low level of trophocyte ploidy in basal infraorders (Nepomorpha and Gerromorpha) and a high such level in the more advanced Pentatomomorpha. Data on additional species of the same and other infraorders are needed to confirm this tendency.
